# Pathogenesis of Frontotemporal Lobar Degeneration: Insights From Loss of Function Theory and Early Involvement of the Caudate Nucleus

**DOI:** 10.3389/fnins.2018.00473

**Published:** 2018-07-12

**Authors:** Gen Sobue, Shinsuke Ishigaki, Hirohisa Watanabe

**Affiliations:** Nagoya University Graduate School of Medicine, Brain and Mind Center, Nagoya University, Nagoya, Japan

**Keywords:** frontotemporal lobar degeneration, loss of function, tau proteins, fused in sarcoma/translated in liposarcoma, amyotrophic lateral sclerosis, TDP-43 proteinopathies, caudate nucleus, marmoset

## Abstract

Frontotemporal lobar degeneration (FTLD) is a group of clinically, pathologically and genetically heterogeneous neurodegenerative disorders that involve the frontal and temporal lobes. Behavioral variant frontotemporal dementia (bvFTD), semantic dementia (SD), and progressive non-fluent aphasia (PNFA) are three major clinical syndromes. TDP-43, FUS, and tau are three major pathogenetic proteins. In this review, we first discuss the loss-of-function mechanism of FTLD. We focus on FUS-associated pathogenesis in which FUS is linked to tau by regulating its alternative splicing machinery. Moreover, FUS is associated with abnormalities in post-synaptic formation, which can be an early disease marker of FTLD. Second, we discuss clinical and pathological aspects of FTLD. Recently, FTLD and amyotrophic lateral sclerosis (ALS) have been recognized as the same disease entity; indeed, nearly all sporadic ALS cases show TDP-43 pathology irrespective of FTD phenotype. Thus, investigating early structural and network changes in the FTLD/ALS continuum can be useful for developing early diagnostic markers of FTLD. MRI studies have revealed the involvement of the caudate nucleus and its anatomical networks in association with the early phase of behavioral/cognitive decline in FTLD/ALS. In particular, even ALS patients with normal cognition have shown a significant decrease in structural connectivity between the caudate head networks. In pathological studies, FTLD/ALS has shown striatal involvement of both efferent system components and glutamatergic inputs from the cerebral cortices even in ALS patients. Thus, the caudate nucleus may be primarily associated with behavioral abnormality and cognitive involvement in FTLD/ALS. Although several clinical trials have been conducted, there is still no therapy that can change the disease course in patients with FTLD. Therefore, there is an urgent need to establish a strategy for predominant sporadic FTLD cases.

## Introduction

Frontotemporal lobar degeneration (FTLD) is a clinically, genetically, and pathologically heterogeneous neurodegenerative disorder that causes selective neuronal loss and gliosis of the frontal and temporal lobes of the brain (Olney et al., 2017). “FTLD” is applied to patients whose diagnoses are genetically or pathologically confirmed. “Frontotemporal dementia (FTD)” is applied to patients who have clinicoradiological abnormalities that correlate with FTLD but are not genetically or pathologically confirmed. Clinically, FTD patients exhibit progressive changes in behavior, language, and executive control. TAR DNA–binding protein 43 kDa (TDP–43), fused in sarcoma/translated in liposarcoma (FUS/TLS), and tau are three major causative proteins in FTLD. TDP-43 and FUS are classified as RNA-binding proteins. Interestingly, these proteins are associated with similar and consecutive clinicoradiological phenotypes.

Clarifying the upstream process of neurodegeneration and developing biomarkers for early diagnosis are essential for the future development of disease-modifying therapy for FTLD. In Asian countries, including Japan, familial FTLD is very rare (accounts for less than 10% of FTLD) (Fukuhara et al., 2014). In Western countries, more than half of FTLD patients have no family history of the disorder, although approximately 40% of patients have a familial trait. However, imaging and biological markers for sporadic FTLD, particularly those in prodromal cases, are very limited compared with the number available for Alzheimer’s disease (AD) (Olsson et al., 2016) and dementia with Lewy bodies (DLB) (Iranzo et al., 2016).

Amyotrophic lateral sclerosis (ALS) has traditionally been considered a progressive neurodegenerative disorder in which the motor system is selectively involved (Hardiman et al., 2017). However, some ALS patients also present with the characteristic clinical findings of FTD during the early course of their illness (Tsujimoto et al., 2011; Masuda et al., 2016). Additionally, FTLD patients can also show upper and lower motor symptoms (Riku et al., 2014b). TDP-43, a major component of ubiquitinated inclusions, is a critically important pathogenic protein found in both sporadic FTLD and ALS (Neumann et al., 2006). Because nearly all sporadic ALS cases and more than half of sporadic FTLD cases have cytoplasmic inclusions consisting of the cleaved form of hyper-phosphorylated TDP-43 (Ling et al., 2013), TDP-43 proteinopathy was proposed as a concept that implicates FTLD-TDP and ALS as a continuous disease spectrum.

With respect to familial ALS and FTLD, a non-coding hexanucleotide repeat expansion (GGGGCC) mutation in the 50 non-coding region of the C9orf72 gene identified in familial ALS and FTLD in 2011 has provided important insights into the pathophysiological backgrounds of ALS and FTLD (DeJesus-Hernandez et al., 2011). In Western countries, the C9orf72 repeat expansion is the most common causative gene of familial ALS and FTLD. Up to 40% of patients with familial ALS, 25% with familial FTLD, especially, 88% with familial ALS-FTLD show the pathological repeat expansion (Van Mossevelde et al., 2017). Interestingly, even sporadic ALS and FTLD patients may also have the C9orf72 repeat expansion. Patients with C9orf72 repeat expansion are classified as TDP-43 pathology spectrum but show heterogeneous clinical and pathological characteristics. There are other major causal ALS genes such as SOD1, TARDBP, FUS, VCP, and PFN1. More recently, advancement of massive parallel sequencing approaches identified rare genetic variants (TBK1, CHCHD10, TUBA4A, CCNF, MATR3, NEK1, C21orf2, ANXA11, TIA1) (Nguyen et al., 2018). Pathogenic mutations of C9orf72, GRN, TBK1, and VCP are commonly linked to TDP-43 pathology. However, affected brain regions and clinical phenotype are highly variable among four mutations and are different from sporadic cases (Van Mossevelde et al., 2018).

In Japan, the frequency of the C9orf72 repeat expansion among ALS patients is much lower than that in Western populations (2/52 = 0.4% in Japan) (Ogaki et al., 2012). Twenty-eight known ALS-related genes were identified in only 3.0% of 251 Japanese sporadic ALS patients by next-generation sequencing (Nakamura et al., 2016). A pathological investigation showed that all consecutive 107 autopsied cases clinically diagnosed as sporadic ALS or progressive muscular atrophy showed TDP-43 pathology (Riku et al., 2014a). Thus, clinical and radiological factors associated with socio-cognitive decline in sporadic ALS may indicate that most sporadic ALS cases are associated with FTLD-TDP-related pathophysiology, although the continuity of the ALS/FTD spectrum remains unknown. Since sporadic cases are predominant in ALS/FTD spectrum even in Western countries, it would be crucial to identify the early structural and network changes associated with socio-cognitive decline in FTLD/ALS.

In this review, we introduce recent findings that can help detect the (1) key pathogenesis leading to neurodegeneration and (2) early structural and network changes related to early diagnosis in FTLD/ALS.

### Loss-of-Function Mechanism of RNA-Binding Proteins in FTLD/ALS

#### Hypothesis of Loss-of-Function Theory in FTLD/ALS

Many genes have been identified in the development of ALS and FTLD, including TDP-43, FUS, and C9orf72, indicating that these two disorders comprise a spectrum of diseases (Seelaar et al., 2011; Renton et al., 2014; Riku et al., 2014b; Hayes and Rothstein, 2016). The cytoplasmic inclusion composing of TDP-43 and FUS in motor and/or cortical neurons is observed in FTLD/ALS as a major pathological hallmark, as observed in other neurodegenerative disorders (Neumann et al., 2006, 2009; Mackenzie and Neumann, 2012). There are two hypotheses for the pathomechanism of RNA-binding proteins such as TDP-43 or FUS: gain of toxicity and loss of function (Xu, 2012; Orozco and Edbauer, 2013). According to the first hypothesis, toxicity from the dislocated and aggregated protein is considered a possible cause of neuronal degeneration. According to the second hypothesis, impaired function of these nuclear proteins provoked by protein dislocation from the nucleus is considered to disturb neuronal function and lead to subsequent neuronal degeneration.

The latter hypothesis is supported by several pieces of evidence, including the fact that TDP-43 or FUS nuclear staining is decreased in the nuclei of neurons in both human FTLD/ALS tissue (Davidson et al., 2007; Neumann et al., 2009) and TDP-43-overexpressing mice (Wegorzewska et al., 2009; Igaz et al., 2011). Moreover, animal models involving the loss of either TDP-43 or FUS mimic the pathology of FTLD/ALS (Kabashi et al., 2011; Wang et al., 2011; Wu et al., 2012; Iguchi et al., 2013; Udagawa et al., 2015). Interestingly, the up- and down-regulation of TDP-43 in Drosophila produced highly similar transcriptome alterations (Vanden Broeck et al., 2013). The gene expression profiles in neurons with simultaneous depletion of FUS and TAF15 were observed to be similar to those in ALS patient-derived neurons bearing the mutation of FUS^R521G^ (Kapeli et al., 2016). These findings indicate that loss of function and/or gain of toxicity of TDP-43 or FUS might influence wide-ranging RNA metabolism pathways in a similar manner.

However, recent evidence indicates that effects of loss of TDP-43 or FUS function on motor neurons are limited. Motor neuron-specific TDP-43 knock-out exhibited mild functional impairments in aged mice without prominent motor neuron loss (Iguchi et al., 2013). Similarly, the absence of FUS in motor neurons did not cause apparent ALS phenotypes in mice (Scekic-Zahirovic et al., 2016; Sharma et al., 2016). Thus, the absence of obvious neurodegeneration by FUS knock-out in motor neurons may indicate that loss of FUS function does not contribute to motor neuron degeneration in ALS. However, FUS knock-down or knock-out in hippocampal neurons and/or frontal lobe neurons exhibited behavioral impairments characterized by lack of anxiety, disinhibition, and hyperactivity but without apparent cognitive impairments in early disease stages (Kino et al., 2015; Udagawa et al., 2015; Ishigaki et al., 2017). Hippocampus-specific FUS silencing results in neuronal loss and hippocampal atrophy in aged mice (Ishigaki et al., 2017). These findings suggest that loss of FUS function in cerebral neurons could contribute to the neuronal dysfunction and neurodegeneration observed in FTLD.

The hippocampus is one of the most strongly affected regions in FTLD, similarly to Alzheimer’s disease. Although the major function of the hippocampus is learning and memory, it has been shown that hippocampal lesions can cause emotional deficits, including hyperactivity, disinhibition, and anxiety (Bannerman et al., 2001; Barkus et al., 2010). A recent study further demonstrated that the specific subregions of the hippocampus are responsible for distinct behaviors; for example, the dorsal and ventral dentate gyri affect memory and anxiety, respectively (Kheirbek et al., 2013). Taken together with findings indicating that loss of FUS leads to neuronal cell death in Zebrafish and Drosophila (Kabashi et al., 2011; Wang et al., 2011), loss of FUS function in the hippocampus can explain the clinical and pathological features of FTLD.

#### Regulation of Tau Isoforms by FUS

Many neuronal function-related genes have been identified to be regulated by FUS (Ishigaki et al., 2012; Lagier-Tourenne et al., 2012; Rogelj et al., 2012; Honda et al., 2013; Nakaya et al., 2013). Intriguingly, FUS itself is auto-regulated by exon 7 skipping machinery that undergoes non-sense-mediated decay (NMD), indicating that the expression of FUS is tightly regulated and that dysregulation by disease-associated mutations is harmful to neurons (Zhou et al., 2013).

We and other groups have identified that FUS regulates an alternative splicing event of the *Mapt* gene at exon 10, which increases 4-repeat tau (4R-tau) but decreases 3-repeat tau (3R-tau) in neurons (Ishigaki et al., 2012; Lagier-Tourenne et al., 2012; Orozco et al., 2012; Rogelj et al., 2012; Fujioka et al., 2013). Loss of FUS functionality is relevant to the pathogenesis of FTLD/ALS by demonstrating that formation of the FUS intranuclear complex with splicing factor, proline-, and glutamine-rich (SFPQ), an RNA-binding protein, in neurons is compromised by FTLD/ALS-associated mutations (Ishigaki et al., 2017). SFPQ is involved in RNA splicing, DNA synthesis, gene expression, DNA repair, and cell survival (Shav-Tal and Zipori, 2002; Kameoka et al., 2004). Although FUS also binds to many other splicing factors in the crude nuclear extract, those proteins are mainly enriched in the small MW FUS complex of two FUS complexes with high MW and small MW. Thus, the high MW FUS complex and SFPQ are thought to compose the splicing machinery (Ishigaki et al., 2017). A recent study revealed novel variants of the SFPQ gene in familial ALS cases (Thomas-Jinu et al., 2017), supporting the idea that the quality loss of FUS function might be a key causal or pathological factor in FTLD/ALS. It has been previously proposed that a compromised RNA-binding protein machinery complex in the nucleus could be a cause of TDP-43- or FUS-associated FTLD/ALS (Tsuiji et al., 2013; Sun et al., 2015). Given that various RNA-binding proteins are genetically and pathologically linked to FTLD/ALS (Ling et al., 2013), our results also strengthen the hypothesis that functional disturbance of RNA metabolism in the nucleus of neurons is crucial for FTLD/ALS pathogenesis.

Because many splicing events are species-specific, it is necessary to confirm that the alternative splicing event for tau isoforms is regulated by FUS and SFPQ in human brain tissues. *MAPT* encodes tau protein, which is pathogenic for tauopathies, such as Alzheimer’s disease and FTLD, both of which are characterized by an accumulation of phosphorylated tau in affected neurons. Pathologically, it is reported that a high 4R-tau/3R-tau ratio is observed in tauopathies, including FTLD (Hong et al., 1998; Yoshida, 2006; Umeda et al., 2013). Therefore, the impaired tau isoform ratio is likely to occur due to the dysregulation of the alternative splicing caused by dysfunction of the FUS-SFPQ complex in the pathogenesis of FTLD/ALS (Ishigaki et al., 2017). A recent case report of atypical FTLD family cases characterized by astrocyte-predominant tauopathy, an aberrant tau isoform ratio, and a Q140H substitution in the FUS gene (Ferrer et al., 2015) supports the notion of a pathophysiological link between FUS and tau in FTLD/ALS through the regulation of 4R-tau/3R-tau isoforms. Further pathological investigation is needed to clarify the role of FUS/SFPQ in the pathogenesis of FTLD/ALS and tauopathies.

#### Regulation of Synapse-Related Genes by FUS

**FIGURE 1:**
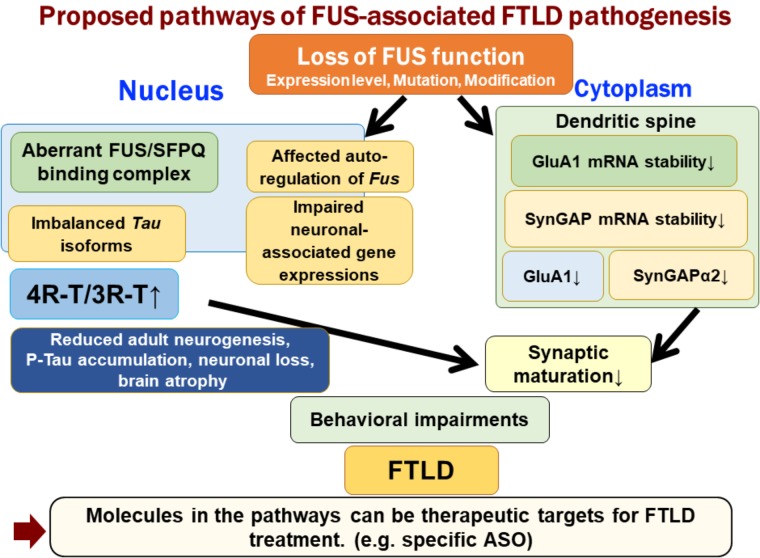
Proposed pathways of FUS-associated FTLD pathogenesis. Loss of FUS function is involved in the early phenotypic pathomechanism of FTLD/ALS. Loss of FUS function in the nucleus, such as aberrant interaction with SFPQ, causes an imbalance in tau isoforms, which subsequently affects adult neurogenesis, phosphorylated tau accumulation, and neurodegeneration (Ishigaki et al., 2017). However, loss of FUS in the dendritic spine reduces the stability of GluA1 and SynGAP mRNA. This synaptic protein reduction impairs the maturation of spines (Udagawa et al., 2015; Yokoi et al., 2017). The two FUS-mediated molecular pathways lead to behavioral impairments, including emotional defects mimicking those observed in FTLD patients.

FUS is an RNA-binding protein that is mainly located in the nucleus; however, it also occurs in neuronal processes. It has been reported that loss of FUS could lead to abnormal synaptic function and morphology (Fujii and Takumi, 2005; Aoki et al., 2012). FUS regulates the expressions of GluA1, a subunit of α-amino-3-hydroxy-5-methyl-4-isoxazolepropionic acid (AMPA) receptor, and SynGAP1 in the dendritic spines, and loss of FUS affects the function of dendritic spines and subsequent behavioral impairments (Udagawa et al., 2015; Yokoi et al., 2017). FUS binds GluA1 mRNA in the vicinity of the 3′ terminus and maintains its stability, likely via the control of poly(A) tail maintenance. GluA1 reduction upon FUS knock-down affects AMPA receptor surface expression and synaptic transmission in neurons (Udagawa et al., 2015). Similarly, FUS regulates the expression of SynGAP isoform α2, which is critical for spine maturation and cognitive behavior. FUS cooperates with ELAVL and regulates SynGAP mRNA stability at its 3′UTR, resulting in a specific SynGAP isoform expression in a 3′UTR length-dependent manner (Yokoi et al., 2017). Moreover, the imbalance in tau isoforms and the increase in total amount of tau by loss of FUS/SFPQ can cause a redistribution of tau in the dendritic neuritis, which is also observed in tau transgenic models; thus, impairments in the post-synapse caused by FUS depletion might be a key pathogenesis leading to neurodegeneration (**Figure 1**).

### Early Structural and Network Changes in FTLD/ALS Focusing on Caudate Nucleus

#### Caudate Nucleus and FTLD/ALS

Detection of early structural and network changes associated with behavioral and cognitive abnormality in FTLD/ALS is crucial for the development of disease-modifying therapy. Halabi et al. showed that patients with bvFTD had 20 and 23% lower caudate volumes on MRI than did healthy controls and individuals with AD, respectively (Halabi et al., 2013). A different group also found that MRI showed a significant volume reduction in the caudate (left 16%, right 11%) in a bvFTD group compared with that observed in controls (Macfarlane et al., 2015). In another study, 30% of FTD cases showed a significant reduction in FP-CIT SPECT uptake in the putamen and the caudate (Morgan et al., 2012). Atrophic changes of the caudate nucleus were also reported to be evident prior to extensive cortical changes in ALS-FTD patients (Masuda et al., 2016). Pathological results showed that even ALS patients exhibited significant efferent neuron loss in the caudate nucleus (Riku et al., 2016). Thus, the caudate nucleus may be primarily involved in the early phase of FTLD/ALS and associated with cognitive involvement in FTLD/ALS.

The caudate nucleus is associated with not only motor processes but also various non-motor functions, including set-shifting, planning cognitive self-initiated actions, rule learning, action contingency, and bilingualism (Provost et al., 2015). In particular, the impairment of inhibitory control, decision making, and the reward system are impaired in patients with FTLD. Thus, there is no contradiction regarding whether primary caudate nucleus lesions cause various behavioral disorders. Additionally, the caudate nucleus has widespread connections with the medial frontal cortex, anterior cingulate cortex, dorsolateral prefrontal cortex, lateral orbitofrontal cortex, and insular, all of which are affected in FTLD. Thus, mild but widespread involvement of the frontotemporal cortex may be associated with caudate nucleus abnormality. These primary and secondary changes may explain why the caudate nucleus is one of the most vulnerable subcortical structures and why lesions in this region are associated with cognitive decline in FTLD/ALS.

Interestingly, the caudate nucleus is involved in patients with not only FTLD-TDP but also FTLD-FUS and FTLD-tau (Kim et al., 2007; Seelaar et al., 2010; Bradfield et al., 2017). Although it is still unclear whether the caudate nucleus is the most vulnerable region in the context of cognitive impairment, there are some reports that atrophic changes in the caudate nucleus could be more prominent in patients with FTLD-tau and FTLD-FUS than in those with FTLD-TDP. ALS patients who were C9orf72 repeat expansion negative also showed significant volume reductions in the left caudate nucleus, left hippocampus, and right accumbens nucleus compared with healthy controls (Bede et al., 2013; Machts et al., 2015).

#### Caudate Nucleus Involvement Is an Early Event in Relation to Cognitive Decline in FTLD/ALS (**Figure 2**)

**FIGURE 2:**
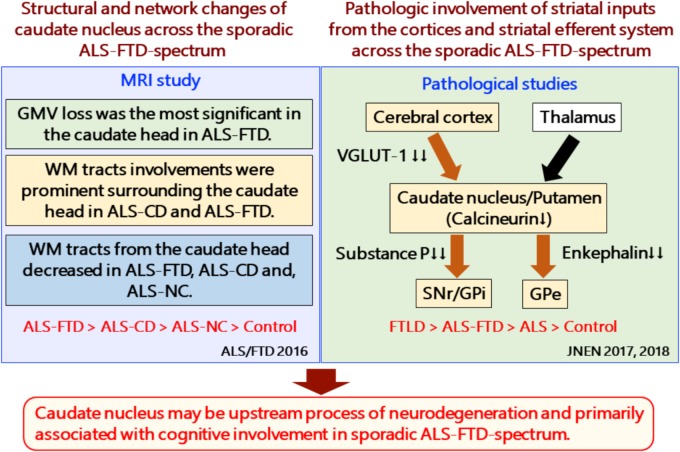
Structural and network changes and pathologic involvement of glutamatergic striatal inputs from the cortices and striatal efferent system across the sporadic ALS-FTD spectrum. MRI studies demonstrated structural and network changes of caudate nucleus across the sporadic ALS-FTD-spectrum as follows: (1) Gray matter volume loss was the most significant in the caudate head in ALS-FTD patients relative to controls. (2) TBSS analysis showed significantly decreased FA values in widespread white matter, particularly surrounding the caudate nucleus in patients with ALS-CD and ALS-FTD, relative to controls (3) Probabilistic diffusion tractography from the head of the caudate nucleus showed extensive decreased connectivity in not only ALS-FTD and ALS-CD but also in ALS-NC. FTLD-TDP patients showed a significant reduction in the axon terminals of the glutamatergic cortical-striatal projection neurons at the caudate head and putamen, as quantified by VGLUT-1 immunohistochemistry. ALS patients showed decreased loss of VGLUT-1-positive axon terminals predominantly in the putamen. CN-positive efferent neurons from the striatum were markedly involved in the following order: ALS < FTD-MND < FTD. The striatal neuronal loss was more predominant in the caudate head than in the putamen. All FTLD-TDP patients exhibited a significant reduction in axon terminals of striatal efferent neurons immunohistochemically assessed by substance-P and enkephalin in the SNr/GPi and GPe. In particular, losses of substance-P-positive projections to the SNr and GPi were consistently severe. Similar findings were obtained in ALS-TDP patients but were mild to moderate. ALS-NC, ALS- normal cognitive; ALS-CD, ALS-cognitive deficiency; ALS-FTD, ALS with frontotemporal dementia; FTD, frontotemporal dementia; FWEc, family-wise error corrected at the cluster level for multiple comparisons; FA, fractional anisotropy; RD, radial diffusivity; TFCE, threshold-free cluster enhancement; TBSS, tensor-based spatial statistics. FTLD-TDP, TAR DNA-binding protein-43 kDa-related frontotemporal lobar degeneration (FTLD-TDP); ALS, amyotrophic lateral sclerosis; FTD, frontotemporal dementia; FTD-MND, FTD with motor neuron disease; VGLUT-1, anti-vesicular glutamate transporter-1; SNr, nigra pars reticulate; GP, globus pallidus (GP); GPi, internal segment of GP; GPe, external segment of GP; CN, calcineurin.

MR volumetry can provide common structural changes across the sporadic ALS and ALS-FTD continuum. Recent studies showed that patients with ALS could have widespread basal ganglia involvement (Bede et al., 2013), in accordance with neuropsychological impairments (Machts et al., 2015). Compared with controls, ALS-FTD showed atrophic changes in the following order of severity: caudate head, medial frontal gyrus, thalamus, amygdala, putamen, and cingulate gyrus. The caudate head atrophy was still significant at the cluster level using Family Wise Error (FWE) correction (*p* < 0.05) (Masuda et al., 2016).

Anatomical network analysis by diffusion tensor imaging with tract-based spatial statistics (TBSS) showed white matter involvements in the areas surrounding the caudate head, the internal capsule, and the anterior horn of the lateral ventricle in patients with ALS with cognitive deficiency (ALS-CD) and ALS-FTD (Masuda et al., 2016). Interestingly, probabilistic diffusion tractography showed a significant decrease in structural connectivity between the caudate head and the dorsomedial frontal cortex and the lateral orbitofrontal cortex in patients with not only ALS-CD and ALS-FTD but also ALS with normal cognitive function.

#### Striatal Efferent System Components in FTLD/ALS

According to recent pathological studies, patients with FTLD-TDP and ALS-TDP demonstrate frequent neuronal loss and/or TDP-43 inclusions in the striatum. In a study that investigated the striatal efferent system using 59 consecutively autopsied patients with sporadic FTLD-TDP or ALS-TDP (Riku et al., 2016), all patients showed markedly reduced striatal medium spiny neurons (efferent neurons) labeled by anti-calcineurin immunohistochemistry prominently in the caudate head. ALS patients revealed mild neuronal loss in the caudate head.

The axon terminals of striatal efferent neurons were immunohistochemically assessed in the substantia nigra pars reticulata (SNr) and globus pallidus interna (GPi) and externa (GPe). All patients with FTLD-TDP showed a distinct reduction in axon terminals in the SNr, GPi, and GPe, regardless of the duration of the illness. In particular, substance-P-positive projections to the SNr and GPi consistently showed severe depletion. Approximately 69.0% of the ALS-TDP patients showed similar but mild involvements compared with FTLD-TDP patients. A significant accumulation of phosphorylated TDP-43 was observed in striatal efferent neurons, efferent tracts, or their axon terminals in the SNr, GPi, and GPe in both FTLD-TDP and ALS-TDP.

These results indicate that there is a strong association between the severity of striatal efferent system involvement and the development of clinical FTD, suggesting that striatal efferent changes parallel the decline of socio-cognitive performance in sporadic FTLD-TDP patients.

#### Glutamatergic Inputs From the Cerebral Cortices Involvement in FTLD/ALS

Glutamatergic inputs from the cerebral cortices to the striatum were also exclusively involved in sporadic FTLD/ALS-TDP. Riku et al. demonstrated that striking depletions of vesicular glutamate transporter-1 (VGLUT-1)-positive axon terminals in the caudate head and putamen were observed in all 46 FTLD-TDP patients examined (31 with FTLD-TDP and 15 with ALS-TDP) (Riku et al., 2017). The ALS-TDP patients involved in the study also showed decreased VGLUT-1-positive axon terminals in the putamen, but those terminals were relatively spared in the caudate head.

A reduction in VGLUT-1-positive axon terminals could also result from a depletion of striatal neuronal density. However, an immunohistochemistry study did not show any association between changes in the expression of VGLUT-1 and synaptophysin. Thus, the severity of the involvement of both caudate nucleus predominant striatal efferent projections and striatal inputs from the cerebral cortices will become evident in association with the impairment in socio-cognitive performance in the TDP-43 proteinopathy spectrum.

Interestingly, confocal microscopy showed that phosphorylated TDP-43 accumulated in the VGLUT-1-positive axon terminals in the striatum of FTLD-TDP and ALS-TDP patients, indicating that aggregations of phosphorylated TDP-43 may play some role in the destruction of VGLUT-1-positive terminals.

#### Relationship Between Caudate Nucleus and Sequential Disease Spreading Demonstrated by Pathologically and Radiologically in FTLD/ALS

Many proteins associated with neurodegenerative diseases, such as TDP-43, tau, and a-synuclein, can show a stereotypical sequential distribution pattern with progression of the disease. Although subcortical structure analysis has not been fully investigated, cortical neural cytoplasmic inclusions in FTLD-TDP, FTLD-FUS, and FTLD-tau share a similar spatial pattern in the frontal and temporal cortex consistent with a “prion-like” spread of pathological proteins (Armstrong, 2017).

Pathological studies have reported evidence of sequential disease spreading in FTLD/ALS. ALS may disseminate in a sequential regional pattern during four disease stages: stage 1, phosphorylated TDP-43 (pTDP-43) pathology involving the agranular motor neocortex—Brodmann areas 4, 6, medulla oblongata at the level of nerve XII—bulbar somatomotor neurons of nerve XII, spinal cord layer 9—ventral horn a-motoneurons; stage 2, prefrontal neocortex (middle frontal gyrus), brainstem reticular formation, precerebellar nuclei, and red nucleus involvements; stage 3, striatum, gyrus rectus, and orbital gyri; and stage 4, anteromedial portions of the temporal lobe, including the hippocampus (Brettschneider et al., 2013).

This pTDP-43 staging pattern has recently been validated *in vivo* using a tract of interest (TOI)-based fiber-tracking approach. TOI analysis demonstrated sequential involvements of the corticospinal tract, the corticorubral and corticopontine tracts, the corticostriatal pathway, and the proximal portion of the perforant pathway in accordance with stages 1, 2, 3, and 4 in ALS (Kassubek et al., 2014). ALS patients with disinhibited behavior showed involvements of seed coordinates of the conticostriatal pathway located in the caudate head or higher (95% specificity and 100% sensitivity) (Lulé et al., 2018), suggesting a disconnect between the caudate head and prefrontal cortex associated with behavioral abnormality in ALS.

Similar sequential distributions of pTDP-43 pathology were also reported in bv FTD patients with TDP-43 Type A or Type B pathology: stage 1, orbital and amygdala; stage 2, additional frontal, temporal, and subcortical regions; stage 3, motor system involvement; and stage 4, visual cortex (Brettschneider et al., 2014). Longitudinal TOI analysis in FTLD also demonstrated a pattern of white matter pathway alterations consistent with patterns of pTDP-43 pathology: stages 1, uncinate fascicle; stage 2, corticostriatal pathway; stage 3, corticospinal tract; and stage 4, optic radiation (Kassubek et al., 2018).

However, whether all patients with ALS and bvFTD can show such a stereotypical sequential distribution pattern throughout the stages of pathology is yet to be determined. The greatest overlap in deposition of TDP-43 between ALS and bvFTD is observed in patients with type B TDP-43 pathology but not in those with type C (Burrell et al., 2016). Even in those with type B, the spreading patterns vary between ALS and bvFTD. Although it may still be difficult to explain various clinical phenotypes and disease progressions based on stereotypical pathological progression, such a propagation theory provides several important insights into the early structural and network changes that occur in ALS and FTLD. The association between behavioral abnormalities and involvement of the corticostriatal tract seeded from the caudate head in ALS and bvFTD supports the view that the caudate head and its network can be the most important target for developing disease-modifying therapy.

### Future Direction

A line of recent studies has suggested that loss of FUS and/or TDP-43 might contribute to early clinico-pathogenesis of FTLD/ALS. Pathological and imaging studies have also revealed that the caudate nucleus is the target candidate for early differential diagnosis between FTLD and AD. Because the caudate nucleus and the putamen are not distinguishable in rodents, it would be suitable to use non-human primates to investigate the pathophysiological relevancy of the caudate and FTLD.

Recently, an FTLD marmoset model was generated by silencing FUS gene using AAV encoding shRNA against the marmoset FUS gene (shFUS). The AAV encoding shFUS (AAV-shFUS) was introduced into the frontal cortex of young adult marmosets by stereotaxic injection, which enabled FUS expressions to be reduced by approximately 70–80%, with an increase in astrocytes and microglias (Endo et al., 2017). The non-human primate FTLD models of caudate-specific FUS-silencing may provide useful information about the importance of a loss-of-function model and the caudate nucleus in FTLD.

Further studies on animal behaviors, imaging studies, and clinic-pathological studies of patients are necessary to establish the caudate nuclei as an early-stage biomarker of FTLD.

## Conclusion

In this review, we demonstrated the loss-of-function mechanism of FTLD, focusing on FUS-associated pathogenesis. Interestingly, FUS was linked to tau by regulating its alternative splicing machinery and was associated with abnormalities in post-synaptic formation that are expected to be an early disease marker of FTLD. We also introduced the involvement of the caudate nucleus and its network in FTLD/ALS, which is believed to be the key to determining the pathogenesis leading to neurodegeneration. We believe that elucidation of the upstream process of neurodegeneration and the development of imaging biomarkers for early diagnosis will allow for the future development of disease-modifying therapy for FTLD.

## Author Contributions

GS, SI, and HW: conceptualization, formal analysis, investigation, methodology, resources, visualization, writing – original draft, and writing – review and editing. SI and HW: data curation. GS: funding acquisition and supervision.

## Conflict of Interest Statement

The authors declare that the research was conducted in the absence of any commercial or financial relationships that could be construed as a potential conflict of interest.
